# Formula Diet Alters the Ileal Metagenome and Transcriptome at Weaning and during the Postweaning Period in a Porcine Model

**DOI:** 10.1128/mSystems.00457-20

**Published:** 2020-08-04

**Authors:** Ahmed A. Elolimy, Charity Washam, Stephanie Byrum, Celine Chen, Harry Dawson, Anne K. Bowlin, Christopher E. Randolph, Manish K. Saraf, Laxmi Yeruva

**Affiliations:** a Arkansas Children’s Nutrition Center, Little Rock, Arkansas, USA; b Department of Pediatrics, University of Arkansas for Medical Sciences, Little Rock, Arkansas, USA; c Department of Biochemistry and Molecular Biology, University of Arkansas for Medical Sciences, Little Rock, Arkansas, USA; d Diet, Genomics & Immunology Laboratory, USDA-ARS Beltsville Human Nutrition Research Center, Beltsville, Maryland, USA; e Arkansas Children’s Research Institute, Little Rock, Arkansas, USA; USDA—Agricultural Research Service, Boyce Thompson Institute, Cornell University

**Keywords:** breast milk, formula milk, ileal RNA-seq, immune response, inflammation, mucosa microbiota, neonates

## Abstract

Exclusive breastfeeding impacts the intestinal microbiome and is associated with a better immune function than is seen with milk formula (MF) feeding in infants and yet with mechanisms poorly defined. The porcine model was used to evaluate the impact of MF on ileum microbial communities and gene expression relative to human milk (HM)-fed piglets. Fifty-two Dutch Landrace male piglets were fed an isocaloric diet of either HM (*n* = 26) or MF (*n* = 26) from day 2 through day 21 of age and weaned to a solid diet until day 51. Eleven piglets from each group were euthanized at day 21, while the remaining piglets (HM, *n* = 15; MF, *n* = 15) were euthanized at day 51 to collect ileal epithelium (EP) scrapings and ileal (IL) tissues. The epithelial mucosa was subjected to shotgun metagenome sequencing, and EP and IL tissues were used for transcriptome analysis. On day 21, transcriptome data revealed that the levels of pathways involved in inflammation and apoptosis were significantly higher in MF piglets than in HM piglets, whereas the levels of tight junctions and pathogen detection systems were lower in MF piglets than in HM piglets. The MF impacts on the small intestine were maintained over the postweaning period (day 51) as indicated by higher levels of Dialister invisus bacteria and higher levels of expression of genes associated with inflammation and apoptosis pathways relative to HM group. The current study demonstrated that MF might impact local intestinal inflammation, apoptosis, and tight junctions and might suppress pathogen recognition in the small intestine compared with HM.

**IMPORTANCE** Exclusive human milk (HM) breastfeeding for the first 6 months of age in infants is recommended to improve health outcomes during early life and beyond. When women are unable to provide sufficient HM, milk formula (MF) is often recommended as a complementary or alternative source of nutrition. Previous studies in piglets demonstrated that MF alters the gut microbiome and induces inflammatory cytokine production. The links between MF feeding, gut microbiome, and inflammation status are unclear due to challenges associated with the collection of intestinal samples from human infants. The current report provides the first insight into MF-microbiome-inflammation connections in the small intestine compared with HM feeding using a porcine model. The present results showed that, compared with HM, MF might impact immune function through the induction of ileal inflammation, apoptosis, and tight junction disruptions and likely compromised immune defense against pathogen detection in the small intestine relative to piglets that were fed HM.

## Abstract (precis)

Exclusive human milk (HM) breastfeeding for the first 6 months of age in infants is recommended to improve health outcomes during early life and beyond. When women are unable to provide sufficient HM, milk formula (MF) is often recommended as a complementary or alternative source of nutrition. Previous studies in piglets demonstrated that MF alters the gut microbiome and induces inflammatory cytokine production. The links between MF feeding, gut microbiome, and inflammation status are unclear due to challenges associated with the collection of intestinal samples from human infants. The current report provides the first insight into MF-microbiome-inflammation connections in the small intestine compared with HM feeding using a porcine model. The present results showed that, compared with HM, MF might impact immune function through the induction of ileal inflammation, apoptosis, and tight junction disruptions and likely compromised immune defense against pathogen detection in the small intestine relative to piglets that were fed HM.

## INTRODUCTION

Human milk (HM) is the cornerstone of early infant nutrition, and HM feeding is associated with several positive health outcomes in children (1, 2). The American Academy of Pediatrics (AAP) recommends exclusive HM feeding during the first 6 months of infancy (1). When women are unable to exclusively feed HM, the AAP recommends providing cow’s milk formula (MF) as a nutritional alternative to HM (1). The National Health and Nutrition Examination Survey (NHANES) reported that more than 81% of infants in the United States between 0 to 12 months of age consumed some formula (3). Several studies showed that MF alters gut microbiome and attenuates immune function in the large intestine compared with the results seen with human milk (HM)-fed piglets (4), because MF does not contain the immune components that HM contains, including anti-inflammatory agents, immunomodulators, and leukocytes (5). For example, human infants fed with cow milk formula had higher levels of *Firmicutes* and lower levels of *Bacteroidetes*, *Enterococcus*, *Streptococcus*, *Enterobacter*, *Lactococcus*, and *Propionibacterium* in the stool than breastfed infants (6). In addition, human infants fed MF showed greater inflammation status than HM-fed infants through the induction of proinflammatory biomarkers such as interleukin-8 (IL-8) and IL-1β in stool (6). Many of these alterations are associated with the development of inflammatory intestinal diseases such as diarrhea, Crohn’s disease, and ulcerative colitis in human infants fed MF (7–9). And yet, the underlying MF-microbiome-gut immunity interactions remain unclear, in part due to the ethical and logistical constraints of obtaining intestinal tissues from human infants (4).

Therefore, studies performed by our group and others have used the porcine model to examine these interactions (4, 10–12) because pig and human intestines are highly similar with regard to early microbiome colonization and metabolic/immune functions (13, 14). For example, a previous study revealed that MF altered ileum development in piglets, with results showing greater mucosal wall thickness and density (12). Our previous study results indicated that the ileal lumen microbiome in piglets fed MF showed increased levels of *Enterobacteriaceae* spp. and decreased levels of *Lactobacillaceae* spp. and *Clostridia* spp. relative to porcine milk-fed piglets (10). However, changes in the microbiome were not associated with ileal gene expression and diet-dependent effects were not dissected due to the housing of the sow group at a farm.

To elucidate the influence of MF diet on the small intestine, a human-milk-fed porcine model was used because of the similarities in the anatomies and physiologies of the digestive tract between pigs and humans (13, 15, 16). Similarly to studies examining 3-month-old human infants, previous studies found that the use of different protein sources such as bovine milk, hydrolyzed bovine milk, and soybean formula did not change intestinal trypsin and chymotrypsin levels or the rate of absorption of nitrogen in the small and large intestine in 3-week-old piglets, which suggested that 3-week-old piglets are equivalent to 3-month-old human infants in that respect (13, 15, 16). The piglet model is relevant to infant nutrition and gut-related outcomes based on previous studies from our team and the published infant literature. In a recent study, we found that the HM group had lower diversity and richness of microbiota across the luminal regions (4), which supports similar findings in human infants who had received HM compared with their counterparts fed MF (17–19). This further supports the notion that HM-fed piglet could be used to study the impact of HM on the gut in human infants. Furthermore, the ability to collect a large portion of tissue from piglets allows researchers to utilize various analytical approaches with the samples. Therefore, piglets are a valuable model to study the influence of nutrition in human infants (14, 20).

We hypothesized that the MF diet induces shifts in ileal mucosal metagenome and transcriptome in piglets relative to HM feeding. To address this hypothesis, we studied changes in ileal mucosal metagenome and transcriptome in male piglets that received either MF or HM at days 21 and 51.

## RESULTS

### Effects of formula feeding on ileal mucosa-associated microbiota.

**FIG 1:**
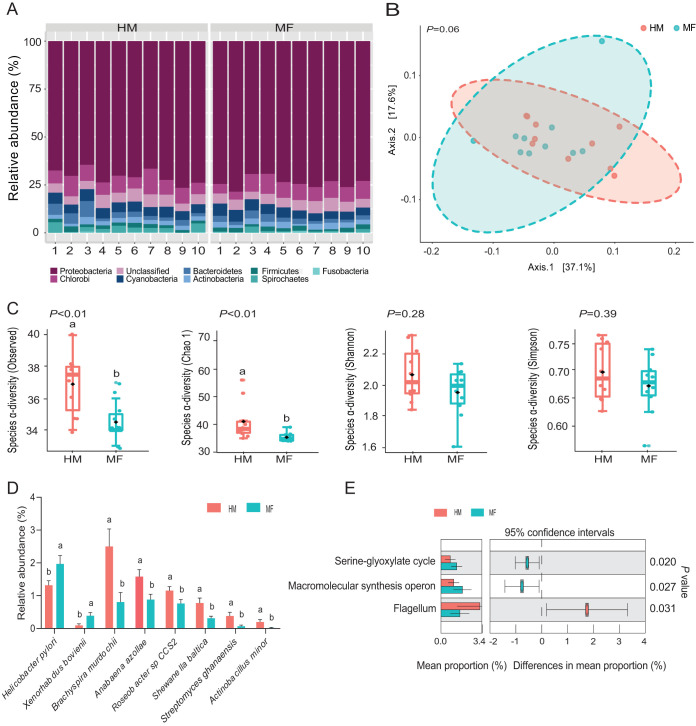
Ileal mucosa-associated bacteria at weaning (i.e., day 21 of age) in male piglets fed human milk (HM, *n* = 10) or milk formula (MF, *n* = 10) from day 2 until day 21 of age. (A) Taxonomic composition of bacterial phyla detected in HM and MF groups. (B) Beta diversity at the bacterial species level determined by principal-coordinate analysis (PCoA). (C) Alpha diversity at the bacterial species level represented by Observed, Chao1, Shannon, and Simpson indices. (D) Significant differences in bacterial species (*P < *0.05) detected between HM and MF groups. (E) Significant differences in predicted metabolic functions (*P < *0.05) for the metagenome gene profile detected between HM and MF groups.

At weaning on day 21 of age, the mean number of paired-end reads was 2,255 per sample with a standard deviation of 651 reads. For α- and β-diversity measures, no differences were noted at the phylum taxonomic level (data not shown). The taxonomic composition of the bacterial phyla (Fig. 1A) showed that the classified bacterial phyla found at weaning were *Proteobacteria* (72.19%), *Chlorobi* (8.04%), *Cyanobacteria* (5.05%), *Bacteroidetes* (3.02%), *Actinobacteria* (2.50%), *Firmicutes* (1.83%), *Spirochaetes* (1.65%), and *Fusobacteria* (0.30%) (Fig. 1A). MF-fed piglets had a lower abundance of the *Spirochaetes* phylum (*P = *0.05) than the HM-fed group (0.80% versus 2.50%) (Table 1). At the species taxonomic level, no differences were detected in community structure as shown by the results of β-diversity principal-coordinate analysis (PCoA) comparing the MF and HM groups (Fig. 1B). The α-diversity analyses showed lower levels of observed (*P < *0.01) and Chao1 (*P < *0.01) indices in the MF group than in the HM group (Fig. 1C). At the bacterial species level, the rate of prevalence in the MF group was higher for Helicobacter pylori (*P = *0.04) and Xenorhabdus bovienii (*P = *0.02) whereas the same group showed decreased rates of prevalence of Brachyspira murdochii (*P = *0.05), Anabaena azollae (*P = *0.03), *Roseobacter* sp. strain *CCS2* (*P = *0.02), Shewanella baltica (*P = *0.03), Streptomyces ghanaensis (*P < *0.01), and Actinobacillus minor (*P = *0.04) (Fig. 1D; see also Data Set S1, sheet 1, in the supplemental material). Metagenome functional analysis (Fig. 1E) revealed that the MF group had a greater number of microbial genes involved in the serine-glyoxylate cycle (*P = *0.02) and macromolecular synthesis operon (*P = *0.03) and a lower number of microbial genes involved in flagellum (*P = *0.03).

**TABLE 1:** Relative abundances of ileal mucosa-associated bacterial phyla detected at weaning (i.e., day 21 of age) in male piglets fed human milk or milk formula during the preweaning period from day 2 until day 21 of age

Phylum	Mean % abundance ± SEM^*a*^		*P* value^*b*^	FDR^*c*^
	HM (*n* = 10)	MF (*n* = 10)		
*Proteobacteria*	70.63 ± 1.17	73.75 ± 0.92	0.09	0.40
*Chlorobi*	8.23 ± 0.74	7.85 ± 0.62	0.94	0.94
Unclassified	5.44 ± 0.36	5.36 ± 0.27	0.85	0.94
*Cyanobacteria*	5.19 ± 0.18	4.92 ± 0.31	0.43	0.86
*Bacteroidetes*	3.51 ± 0.72	2.54 ± 0.31	0.57	0.86
*Actinobacteria*	2.41 ± 0.21	2.60 ± 0.25	0.20	0.59
*Firmicutes*	1.82 ± 0.16	1.85 ± 0.20	0.79	0.94
*Spirochaetes*	2.50 ± 0.53A	0.80 ± 0.29B	0.05	0.40
*Fusobacteria*	0.27 ± 0.06	0.34 ± 0.07	0.49	0.86

a Different capital letters indicate significant differences between human milk (HM) and milk formula (MF) groups (*P *≤ 0.05).

b *P* values were determined by Mann-Whitney test.

c FDR, false-discovery rate.

10.1128/mSystems.00457-20.1 DATA SET S1 (Sheet 1) Read counts of ileal mucosa-associated bacterial species detected at weaning (i.e., day 21 of age) in male piglets fed human milk (HM, *n* = 10) or milk formula (MF, *n* = 10). (Sheet 2) Read counts of ileal mucosa-associated bacterial species detected at day 51 of age in male piglets fed human milk (HM, *n* = 14) or milk formula (MF, *n* = 14). (Sheet 3) Differentially expressed genes (DEGs) in ileal epithelium scrapings (EP) detected at weaning (i.e., day 21 of age) in male piglets fed milk formula (MF, *n* = 10) compared with male piglets fed human milk (HM, *n* = 10) during the preweaning period from day 2 until day 21 of age. (Sheet 4) Differentially expressed genes (DEGs) in ileal tissues (IL) detected at weaning (i.e., day 21 of age) in male piglets fed milk formula (MF, *n* = 10) compared with male piglets fed human milk (HM, *n* = 10) during the preweaning period from day 2 until day 21 of age. (Sheet 5) Differentially expressed genes (DEGs) in ileal epithelium scrapings (EP) detected at day 51 of age in male piglets fed milk formula (MF, *n* = 14) compared with male piglets fed human milk (HM, *n* = 14) during the preweaning period from day 2 until day 21 of age. (Sheet 6) Differentially expressed genes (DEGs) in ileal tissues (IL) detected at day 51 of age in male piglets fed milk formula (MF, *n* = 14) compared with male piglets fed milk human milk (HM, *n* = 14) during the preweaning period from day 2 until day 21 of age. (Sheet 7) Ingenuity Pathway Analysis (IPA) of canonical pathways in ileal epithelium scrapings (EP) showing elevated levels at weaning (i.e., day 21 of age) in male piglets fed milk formula (MF, *n* = 10) compared with male piglets fed human milk (HM, *n* = 10) during the preweaning period from day 2 until day 21 of age. (Sheet 8) Ingenuity Pathway Analysis (IPA) of canonical pathways in ileal epithelium scrapings (EP) showing decreased levels at weaning (i.e., day 21 of age) in male piglets fed milk formula (MF, *n* = 10) compared with male piglets fed human milk (HM, *n* = 10) during the preweaning period from day 2 until day 21 of age. (Sheet 9) Ingenuity Pathway Analysis (IPA) of canonical pathways in ileal epithelium scrapings (EP) showing elevated levels at day 51 of age in male piglets fed milk formula (MF, *n* = 14) compared with male piglets fed human milk (HM, *n* = 14) during the preweaning period from day 2 until day 21 of age. (Sheet 10) Ingenuity Pathway Analysis (IPA) of canonical pathways in ileal epithelium scrapings (EP) showing decreased levels at day 51 of age in male piglets fed milk formula (MF, *n* = 14) compared with male piglets fed human milk (HM, *n* = 14) during the preweaning period from day 2 until day 21 of age. (Sheet 11) Ingenuity Pathway Analysis (IPA) of canonical pathways showing increased levels in ileal tissues (IL) detected at weaning (i.e., day 21 of age) in male piglets fed milk formula (MF, *n* = 10) compared with male piglets fed human milk (HM, *n* = 10) during the preweaning period from day 2 until day 21 of age. (Sheet 12) Ingenuity Pathway Analysis (IPA) of canonical pathways showing decreased levels in ileal (IL) tissues detected at weaning (i.e., day 21 of age) in male piglets fed milk formula (MF, *n* = 10) compared with male piglets fed human milk (HM, *n* = 10) during the preweaning period from day 2 until day 21 of age. (Sheet 13) Ingenuity Pathway Analysis (IPA) of canonical pathways showing increased levels in ileal tissues (IL) detected at day 51 of age in male piglets fed milk formula (MF, *n* = 14) compared with male piglets fed milk human milk (HM, *n* = 14) during the preweaning period from day 2 until day 21 of age. (Sheet 14) Ingenuity Pathway Analysis (IPA) canonical pathways showing decreased levels in ileal tissues (IL) detected at day 51 of age in male piglets fed milk formula (MF, *n* = 14) compared with male piglets fed milk human milk (HM, *n* = 14) during the preweaning period from day 2 until day 21 of age. Download Data Set S1, XLS file, 9.5 MB. Copyright © 2020 Elolimy et al. 2020 Elolimy et al. This content is distributed under the terms of the Creative Commons Attribution 4.0 International license.

**FIG 2:**
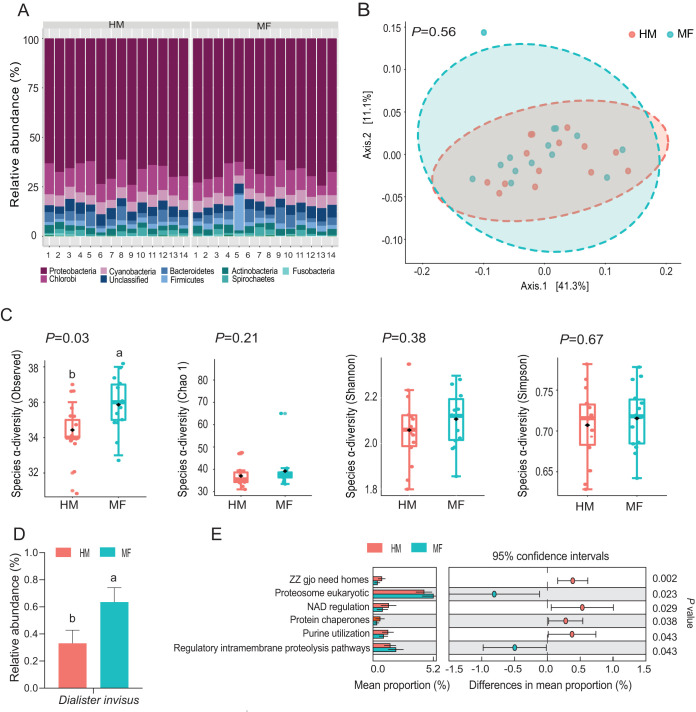
Ileal mucosa-associated bacteria during the postweaning period at day 51 of age in male piglets fed human milk (HM, *n* = 14) or milk formula (MF, *n* = 14) from day 2 until day 21 of age followed by solid diet until day 51. (A) The taxonomic composition of bacterial phyla detected in HM and MF groups. (B) The beta diversity at the bacterial species level analyzed by principal-coordinate analysis (PCoA). (C) The alpha diversity at the bacterial species level analyzed by observed species, Chao1, Shannon, and Simpson indices. (D) Significant differences in bacterial species (*P < *0.05) detected between HM and MF groups. (E) Significant differences in predicted metabolic functions (*P < *0.05) for the metagenome gene profile detected between HM and MF groups.

During the postweaning period at day 51 of age, the mean number of paired-end reads was 2,162 per sample with a standard deviation of 643 reads. No differences in alpha-diversity and beta-diversity were noted at the phylum taxonomic level (data not shown). The taxonomic composition of the bacterial phyla (Fig. 2A) showed that the classified bacterial phyla found at weaning were *Proteobacteria* (67.28%), *Chlorobi* (11.28%), *Cyanobacteria* (5.33%), *Bacteroidetes* (3.84%), *Firmicutes* (2.78%), *Actinobacteria* (2.74%), *Spirochaetes* (1.54%), and *Fusobacteria* (0.32%) (Fig. 2A). The MF group showed increased levels of members of the *Firmicutes* phylum (*P = *0.02) and decreased levels of members of the *Cyanobacteria* phylum (*P = *0.04) compared with HM group (Table 2). At the species taxonomic level, no differences were observed in β-diversity based on PCoA analysis (*P = *0.56) between the MF and HM groups (Fig. 2B). Analyses of α-diversity revealed greater diversity in the MF group than in the HM group as shown by the observed index (*P = *0.03) (Fig. 2C). At the bacterial species level, the members of the MF group displayed higher levels of Dialister invisus (*P = *0.02) (Fig. 2D; see also Data Set S1, sheet 2, in the supplemental material). The metagenomic functional analysis (Fig. 2E) revealed that the MF group had a greater number of microbial genes involved in proteasome eukaryotic pathways (*P = *0.02) and regulatory intramembrane proteolysis pathways (*P = *0.04) whereas the MF group had a lower number of microbial genes involved in ZZ gjo need homes (*P < *0.01), NAD regulation (*P = *0.03), protein chaperones (*P = *0.04), and purine utilization (*P = *0.04).

**TABLE 2:** Relative abundances of ileal mucosa-associated bacterial phyla detected at day 51 of age in male piglets fed human milk or milk formula

Phylum	Mean % abundance ± SEM^*a*^		*P* value^*b*^	FDR^*c*^
	HM (*n* = 14)	MF (*n* = 14)		
*Proteobacteria*	66.79 ± 1.13	67.77 ± 1.03	0.33	0.57
*Chlorobi*	12.25 ± 0.73	10.30 ± 0.77	0.15	0.33
*Cyanobacteria*	5.39 ± 0.15A	5.27 ± 0.20B	0.04	0.18
Unclassified	4.71 ± 0.25	4.72 ± 0.31	0.75	0.75
*Bacteroidetes*	3.97 ± 0.51	3.71 ± 0.57	0.71	0.75
*Firmicutes*	1.99 ± 0.14B	3.57 ± 1.12A	0.02	0.16
*Actinobacteria*	2.69 ± 0.19	2.80 ± 0.21	0.68	0.75
*Spirochaetes*	1.75 ± 0.29	1.34 ± 0.33	0.10	0.31
*Fusobacteria*	0.30 ± 0.10	0.34 ± 0.08	0.38	0.57
*Planctomycetes*	0.18 ± 0.04	0.19 ± 0.04	0.33	0.57

a Different capital letters indicate significant differences between human milk (HM) and milk formula (MF) groups (*P *≤ 0.05).

b *P* values were determined by Mann-Whitney test.

c FDR, false-discovery rate.

### Effects of formula feeding on the transcriptome of ileal epithelium and tissue.

**FIG 3:**
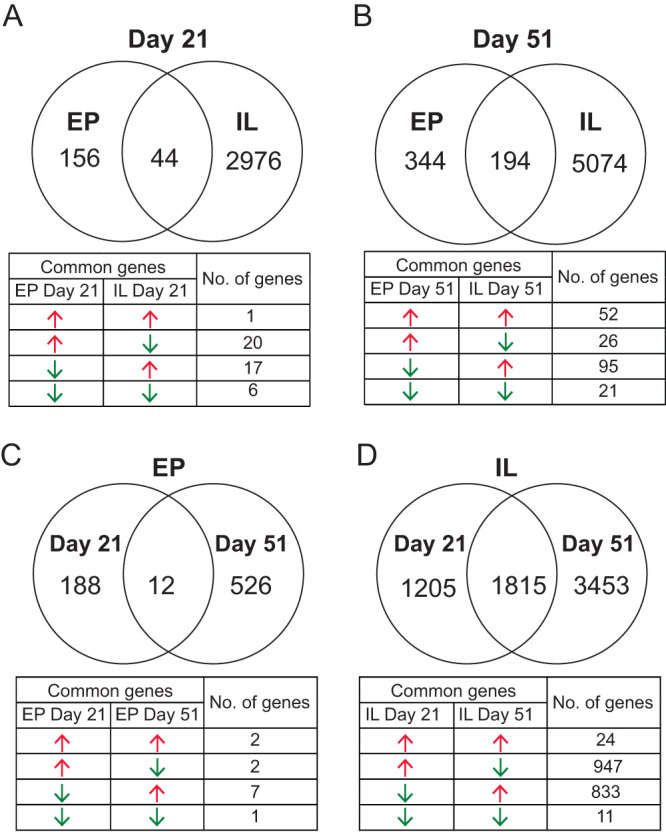
Venn diagram analyses and the overlapped genes distribution table of the differentially expressed genes (DEGs; fold change value of ≥2 and *P* value of ≤0.05) that were identified from the ileal RNA-seq data in male piglets fed milk formula (MF) compared with human milk (HM) fed from day 2 until day 21 of age. (A) Ileal tissues (IL) at day 21 versus ileal epithelium (EP) at day 21 of age. (B) IL versus EP at day 51 of age. (C) IL at day 21 versus at day 51 of age. (D) EP at day 21 versus at day 51 of age. Red arrows indicate increased gene expression, whereas green arrows indicate decreased gene expression in the MF group relative to the HM group.

We assessed the impact of formula feeding on IL and EP global gene expression at day 21 and day 51 of age in piglets using a cutoff *P* value of ≤0.05 and a fold change (FC) value of ≥2 to define differentially expressed genes (DEGs). At day 21, we found 3,176 DEGs in EP and IL of the MF group relative to the HM group (Data Set S1, sheet 3, and Data Set S1, sheet 4, in the supplemental material). Of the total DEGs, 156 were unique to EP and 2,976 were unique to IL in the MF-fed piglets relative to the HM-fed piglets (Fig. 3A). There were 44 common genes between EP and IL. At day 21, among the common DEGs between EP and IL in the MF group, we found that expression of one gene was increased in EP and IL, expression of 6 genes was decreased in EP and IL, expression of 20 genes was increased in EP, and expression of 17 genes was decreased in EP in the MF group relative to the HM group (Fig. 3A). At day 51 of age, 5,612 genes were differentially expressed in EP and IL of the MF-fed group relative to HM-fed piglets (Data Set S1, sheet 5, and Data Set S1, sheet 6, in the supplemental material). In the MF group, 344 of the genes were unique to EP, and 5,074 were unique to IL (Fig. 3B). There were 194 common genes between EP and IL. At day 51, among the common DEGs between EP and IL, 52 genes were increased in EP and IL, while 21 were decreased in EP and IL. Furthermore, 26 were decreased in EP and the same ones were decreased in IL. In EP of the MF group, 95 genes were decreased in EP whereas IL showed increased expression for the same genes in comparison to HM-fed piglets (Fig. 3B).

We compared DEGs between days in EP and IL in MF in comparison to HM. In EP, 188 genes were unique to day 21 and 526 unique to day 51 (Fig. 3C) and 12 were common between day 21 and day 51. We also compared the differentially expressed genes between day 21 and day 51 in IL of MF in comparison to HM. In IL, 1,205 DEGs were unique to day 21 and 3,453 DEGs were unique to day 51 whereas 1,815 were common between day 21 and day 51 (Fig. 3D).

**FIG 4:**
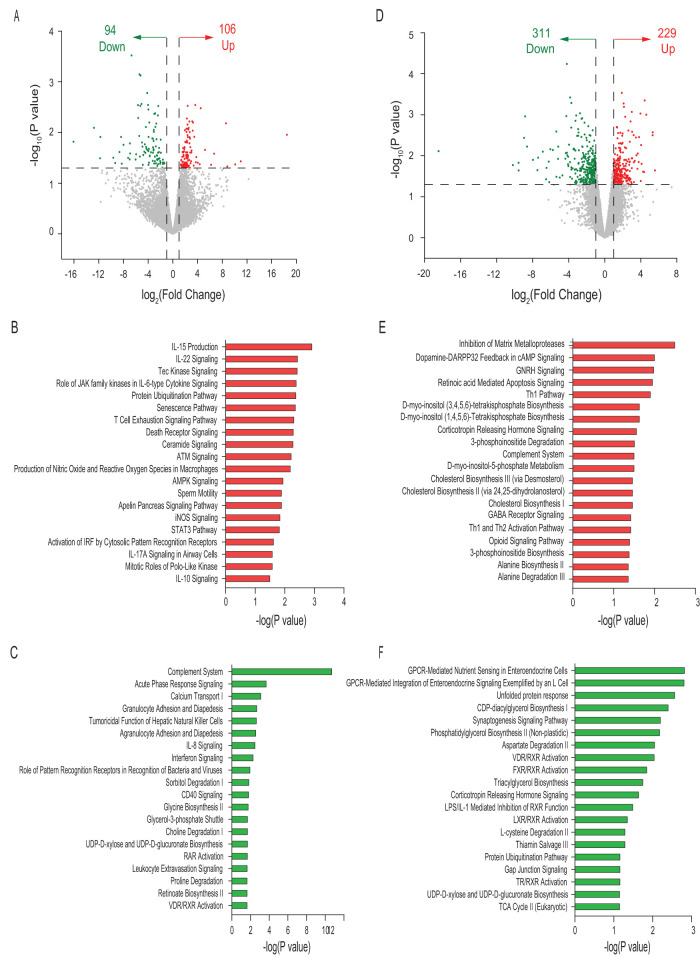
Differential gene expression in ileal epithelium (EP) of male piglets fed milk formula (MF) compared with human milk (HM) from day 2 until day 21 of age. (A) Volcano plot showing genes with decreased (green dots), increased (red dots), and unchanged (gray dots) expression in the MF group at day 21 of age. (B) Top 20 canonical pathways in the MF group showing elevated levels at day 21 of age. AMPK, AMP-activated protein kinase; iNOS, inducible nitric oxide synthase. (C) Top 20 canonical pathways in the MF group showing decreased levels at day 21 of age. (D) Volcano plot showing genes with decreased (green dots), increased (red dots), and unchanged (gray dots) expression in the MF group at day 51 of age. (E) Top 20 canonical pathways in the MF group showing elevated levels at day 51 of age. (F) Top 20 canonical pathways in the MF group showing decreased levels at day 51 of age. TCA, tricarboxylic acid.

The volcano plot of transcriptome sequencing (RNA-seq) data for EP in MF at day 21 showed increased expression of 106 genes and decreased expression of 94 genes in comparison to the HM group (Fig. 4A). The top 20 (Data Set S1, sheet 7, and Data Set S1, sheet 8, in the supplemental material) pathways associated with these genes in the MF group are illustrated in Fig. 4B and D, respectively. The volcano plot for EP at day 51 showed 540 DEGs in the MF group, including increased expression of 229 genes and decreased expression of 311 genes in comparison to the HM group (Fig. 4D). The top 20 pathways associated with these DEG in the MF group relative to the HM group (Data Set S1, sheet 9, and Data Set S1, sheet 10, in the supplemental material) are illustrated in Fig. 4E and F, respectively.

**FIG 5:**
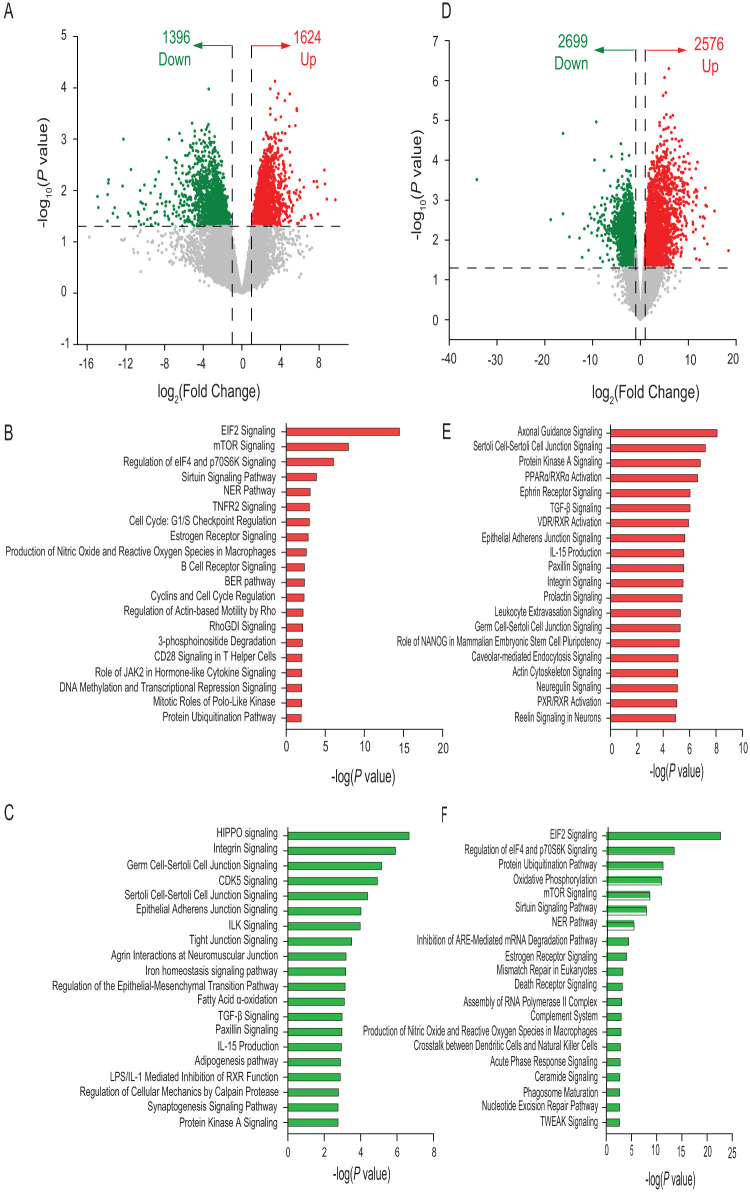
Differential gene expression in ileal tissues (IL) of male piglets fed milk formula (MF) compared with human milk (HM) from day 2 until day 21 of age. (A) Volcano plot showing genes with decreased (green dots) and increased (red dots) expression in the MF group at day 21 of age. (B) Top 20 canonical pathways in the MF group showing elevated levels at day 21 of age. EIF2/eIF2, eukaryotic initiation factor 2; NER, nucleotide excision repair; BER, base excision repair. (C) Top 20 canonical pathways in the MF group showing decreased levels at day 21 of age. (D) Volcano plot showing genes with genes with decreased (green dots), increased (red dots), and unchanged (gray dots) expression in the MF group at day 51 of age. (E) Top 20 canonical pathways in the MF group showing elevated levels at day 51 of age. (F) Top 20 canonical pathways in the MF group showing decreased levels at day 51 of age. TGF-β, transforming growth factor β.

The volcano plot of RNA-seq data for IL at day 21 showed 3,020 DEGs in MF-fed piglets, including higher expression of 1,624 genes and lower expression of 1,396 genes relative to the HM group (Fig. 5A). The top 20 pathways associated with these DEGs in the MF group (Data Set S1, sheet 11, and Data Set S1, sheet 12, in the supplemental material) are illustrated in Fig. 5B and C, respectively. The volcano plot of RNA-seq data for IL at day 51 showed 5,275 differentially expressed genes in the MF group, with genes showing 2,576 higher expression and 2,699 genes showing lower expression than in the HM group (Fig. 5D). The top 20 pathways associated with these DEGs in the MF group (Data Set S1, sheet 13, and Data Set S1, sheet 14, in the supplemental material) are illustrated in Fig. 5E and F, respectively.

## DISCUSSION

Breastfeeding has been shown to have positive health outcomes with respect to immune function and shaping gut microbiota. In our previous study (10), we showed by real-time PCR that a MF diet led to greater ileal inflammation status through increased expression of proinflammatory cytokines (IL-15, IL-8, CCL25, CCL4, CXCL11, FASL, VEGFA, AMCFII, LIF) and downregulation of mRNA of anti-inflammatory cytokines (IL-9, IL-10, IL-27, interferon alpha 4 [IFN-A4], and CSF3) in comparison to piglets fed porcine milk. However, environmental effects might have interfered with the diet effects because piglets fed porcine milk were placed with their sows at the farm during the experimental period. This enabled *ad libitum* breastfeeding, whereas MF piglets were placed with their sows at the farm for only the first 48 h of age and were then moved to the vivarium to feed on MF (Similac Advance powder; Ross Products, Abbott Laboratories, Columbus, OH, USA) from day 2 to day 21 of age (10). In the current study, animals were all housed in the vivarium, allowing a focus on diet-specific effects. To understand the impact of MF feeding versus HM feeding on small-intestine functions at weaning and on the persistence of changes during the postweaning period, we comprehensively evaluated changes in the ileal mucosal metagenome and epithelial cell transcriptome at day 21 and day 51 of age in piglets fed either MF or HM.

### Impact of MF diet relative to HM diet at day 21.

The metagenome data demonstrated that at day 21, MF piglets had higher levels of Helicobacter pylori, a Gram-negative microaerophilic bacterium that colonizes the gastric mucosa and can induce inflammation and mucosal barrier disruption in the gut (21, 22). Therefore, higher levels of colonization of Helicobacter pylori in the MF group might induce a status of higher levels of inflammation and barrier disruptions in the small intestine in formula-fed piglets. Due to the limitations associated with collecting small-intestine contents from human infants, most of the available gut microbiome studies in human infants fed either breast milk or formula milk have used stool samples, which represent the large-intestine microbiome. Whether levels of Helicobacter pylori bacteria increase in the small intestine of human infants fed milk formula compared with breastfed infants is an issue to be addressed. Helicobacter pylori might have transferred from the surrounding environment, including diets and feeders, into the small intestine of HM and MF piglets. The current study did not evaluate the presence of Helicobacter pylori in the housing facilities. However, the higher abundance of Helicobacter pylori in the MF group suggests that the environment in which the MF-fed piglets were maintained might have enhanced Helicobacter pylori colonization in the small intestine.

Pathway analysis revealed that the Tec kinase signaling and cellular senescence pathways were among the top pathways in the MF group EP in the current study whereas levels of eukaryotic initiation factor 2 (EIF2) signaling were higher in IL. Tec kinase and EIF2 signaling and the senescence pathway play a pivotal role in the secretion of proinflammatory cytokines (23–25). In support of this notion, our EP transcriptome data showed that MF induced expression of signaling pathways of proinflammatory cytokines such as IL-15 and IL-22. These proinflammatory cytokines are produced mainly by inflammatory cells, including innate lymphocytes, T cells, and natural killer cells (26, 27), and greatly contribute to the initiation and progression of inflammation status (27–29). Canonical pathway analysis showed that MF reduced the activity of the complement system in EP. The complement system coordinates innate and adaptive immune responses (30). Accumulating evidence has indicated that the complement system is essential for the immune defense system since the complement system regulates the elimination of pathogens through opsonophagocytic mechanisms to maintain host immunosurveillance and tissue homeostasis (31). Downregulation of certain components of the complement pathway has been observed in inflammatory states (32). Canonical pathway analysis also showed that MF lowered the activity of the sorbitol degradation pathway in EP. Tissue sorbitol accumulation can lead to inflammation and cellular dysfunction (33). Several lines of evidence from our group and others have suggested that formula feeding induces local intestinal inflammation in pigs and humans through increased expression of proinflammatory cytokine IL-15 and neutrophil chemoattractants (CXCL6 and IL-8) (6, 10). Another study on infant rhesus monkeys (34) reported that formula feeding elevated levels of serum proinflammatory immune biomarkers, including IL-4, IL-1β, IFN-γ, and tumor necrosis factor α (TNF-α), relative to the breastfeeding group. Most recently, in a rat model, feeding of 2′-fucosal lactose, one of the HMOs (human milk oligosaccharides) present in breast milk, has been shown to reduce inflammatory cytokine production (IL-1β, IL-4, IL-12, IFN-γ, and TNF-α) relative to the results seen with a control group, further highlighting the anti-inflammatory role of breast milk components (35). These results highlight the elevated inflammatory state in formula-fed rhesus infants compared with breast-fed infants (34, 35). Therefore, MF feeding might lead to the induction of tec kinase signaling, senescence pathway, and proinflammatory cytokines and to decreased function of complement system and sorbitol degradation relative to an HM diet.

Previous studies showed that proinflammatory cytokines and ceramides induce apoptosis (36). Our functional analyses of DEGs in EP highlighted the enrichment in the MF group of apoptosis-associated systems such as protein ubiquitination, ATM, ceramide, death receptor signaling, and NER pathways (37–42). In further support of our results, a recent *in vitro* study showed that human milk reduced cellular apoptosis in intestinal epithelial cells through the inhibition of oxidative stress (43). In another study, Xie et al. (44) reported that porcine milk reduced the rate of death of intestinal epithelial cells through the downregulation of p53 and TLR4/NF-κB pathways in piglets. MF lowered the activity of the Hippo signaling cascade. The Hippo signaling pathway plays a pivotal role in the promotion of cell proliferation and the inhibition of cell apoptosis (45). The Hippo signaling cascade also controls organ size in animals by balancing cell growth and death (46). Last, suppression of the activity of the Hippo signaling pathway may serve as a biomarker for apoptosis. In our previous study on piglets fed formula versus piglets fed sow breast milk (47), the predictive function profiling of colon microbiota revealed higher expression of apoptosis-associated pathways in formula-fed piglets (47). Taking the data together, formula feeding might induce cell death and increase the exfoliation of small-intestine epithelial cells in MF, while the mechanisms involved have yet to be determined.

Tight junction proteins are transmembrane proteins that seal off physical barriers and regulate fluid and solute flow through the paracellular space (48). Assembled tight junction proteins provide necessary mechanical linkages, help the formation and maintenance of cellular adhesive contacts, and contribute to cellular organization (49). Disrupted tight junction signaling in mucosa could possibly explain a response to the breakdown of the integrity of epithelial tight junctions (50). Notably, the current study indicated that MF diet reduced the expression of genes involved in key pathways (epithelial adherens junction, integrin, and ILK signaling) that regulate tight junctions in IL (51, 52). Several pathogens invade hosts by taking advantage of the dysregulation of tight junctions in mucosal cells (53). We therefore speculate that this might affect the proper functioning of ileal tight junction molecules in MF. Future studies are warranted to further determine the expression levels and localizations of tight junction proteins to obtain a more comprehensive physiological understanding.

Our data showed that MF suppressed the expression of certain mRNA pattern recognition receptors (PRRs), which participate in the recognition of bacteria and viruses in EP. PRRs include the families of Toll-like receptors (TLRs), RIG-I-like receptors, NOD-like receptors, and C-type lectin receptors (54). It is well established that PRRs are usable for detecting pathogen-associated molecular patterns (55). PRRs signal through a diverse array of intermediate molecular adaptors to activate transcription factors that drive gene transcription responsible for antimicrobial activity (56). Recognition of microbial nucleic acids by endosomal or cytosolic PRRs constitutes a key component in the innate immune system to combat invading bacterial and viral pathogens (57). Based on the findings of the present study, MF might attenuate sensitivity with respect to the elimination of invading pathogens in the small intestine, which suggests that the MF-fed group is at increased risk for pathogenic invasions in the gut. In support of this notion, our previous study on piglets fed formula versus piglets fed porcine breast milk (47) suggested that formula-fed piglets might have a higher chance of epithelial cell invasion by colon bacteria than breast milk-fed piglets (47).

The MF diet reduced expression of genes associated with cyclin-dependent kinase 5 (CDK5) signaling in IL. CDK5 is a proline-directed serine/threonine kinase that plays a critical role in the development and growth of the nervous system; CDK5 is essential for synaptogenesis, neurite outgrowth, neuron migration, axonal guidance, synaptic plasticity, and neurotransmission (58–60). Therefore, it is possible that the MF-mediated decrease of CDK5 signaling in IL might ultimately be attributable to neuronal dysfunction. These results are consistent with the findings of human studies, suggesting that human milk is important for neurodevelopment in infants (61, 62).

### Effects of formula feeding during the postweaning period.

Metagenome data showed that MF piglets had increased colonization of Dialister invisus in ileal mucosa. In a pilot study, Khandelwal at al. (63) reported that 0-to-5-year-old children fed formula versus human milk between day −3 and day +14 around bone marrow transplantation showed increased levels of intestinal Dialister invisus and intestinal inflammation compared with a human milk-fed group. Therefore, higher levels of Dialister invisus in the MF-fed group might lead to intestinal inflammation in cow’s milk formula-fed piglets. In support of this notion, transcriptome data from EP revealed activation of T helper pathways with known inflammation relevance in the MF group such as the Th1 (IL-2, IFN-γ, and TNF-α) and Th2 (IL-4) pathways (64–66). Furthermore, IL transcriptome data revealed the activation of inflammation-involved pathways in the MF group, including IL-15 and ephrin receptor signaling (67, 68). These findings suggest that gut inflammation in MF piglets is likely persistent during the postweaning period.

The EP findings highlighted that the levels of genes associated with apoptosis pathways [retinoic acid-mediated apoptosis signaling, d-myo-inositol (1,4-6)-tetrakisphosphate, and d-myo-inositol (3-6)-tetrakisphosphate biosynthesis] were higher in the MF group. In addition, the current study showed that MF increased the expression of genes involved in transforming growth factor β (TGF-β) signaling in IL, possibly resulting in the suppression of normal epithelial cells growth by induction of cell cycle inhibitors such as p21^CIP1n^ and p15^INK4b^ (69, 70). These data suggest that MF might increase small-intestine apoptosis at weaning.

### Study limitations.

Limitations of the current study included the fact that the human milk fed to piglets represented a pool of donor milk. The interindividual variations of milk composition between donor mothers, including fatty acids (71), metabolites (72), oligosaccharides (73), and microbiota (74), might alter intestinal metagenome and transcriptome. However, the pooled HM used in the current study likely eliminated the impact of interindividual variations of HM on intestinal functions in piglets. The second limiting factor was that the human milk fed to the piglets in the present study was pasteurized. In addition to the destruction of viable microorganisms, several studies previously reported a reduction in levels of human milk micro- and macronutrients and immune components but not protein and human milk oligosaccharides after pasteurization (75–77). Therefore, impact of the pasteurized human milk that was used in the present study might not have exactly mimicked the impact of nonpasteurized human milk offered to breast-fed infants since nutritional content plays a major role in the regulation of intestinal development and function (78). The third limiting factor was that the human milk used was collected from donor mothers at between 2 and 12 months of lactation (average, 6 months). Many previous studies reported changes in milk composition throughout the lactation period. For example, foremilk has lower fat and greater protein content than hindmilk (79, 80). These changes in milk composition might have significant implications for intestinal metagenome and functions. However, pooling the human milk samples collected at different time points between 2 and 12 months of lactation was performed on the basis of the assumption that the changes in milk composition occurring during lactation would not likely affect intestinal development. The fourth limiting factor was associated with the fact that exclusive human breast milk feeding for the first 6 months of age in infants is recommended to improve health outcomes during early life and beyond. However, the current study provided the piglets with a weaning solid starter diet during the preweaning period from day 14 to day 21 alongside HM or MF feeding. Although this liquid-solid diet mixture was provided for both groups, and changes in the intestinal metagenome and transcriptome profiles were most likely due to the impact of HM or MF, it could be that the interaction between the solid diet and HM or MF induced some of these intestinal changes. Future studies are warranted to address this limitation. The whey/casein ratio in human milk dramatically changes during the lactation cycle; it is 80:20 in early lactation and changes to 50:50 later in lactation (81). In contrast, the whey/casein ratio in cow’s milk is 20:80 (82). Therefore, whey protein concentrate is added to the cow milk-based infant formula to make whey/casein ratio similar to the ratio in human milk (83). In the current study, we added whey protein to HM and MF diets to meet the protein requirements for growing pigs according to National Research Council (NRC) guidelines (10). Although we did not evaluate the whey/casein ratios in the current HM and MF diets, we suspect that the supplementation of whey protein most likely increased the whey/casein ratio in the HM and MF diets without any difference in total protein content. Our goal was to formulate a diet to meet the NRC requirements for growing pigs to study the influence of HM or MF on gut functions (4, 10). The only significant difference between the HM and MF diets is that the majority of the one diet is formula and the majority of the other is human milk (see Table S1 in the supplemental material). Therefore, changes in gut functions between the two groups are likely related to the effect of MF versus HM.

10.1128/mSystems.00457-20.2 TABLE S1 Diet composition of milk formula, human milk, and sow milk. Download Table S1, DOCX file, 0.02 MB. Copyright © 2020 Elolimy et al. 2020 Elolimy et al. This content is distributed under the terms of the Creative Commons Attribution 4.0 International license.

In conclusion, the current study highlighted that MF feeding alters metagenome and transcriptome profiles in the mucosal epithelium and ileum tissue relative to a human milk group at weaning and during the postweaning period in piglets. These changes included transcripts potentially reflective of higher inflammation levels, apoptosis, tight junction disruptions, and likely the suppression of immune defense against pathogen recognition in the small intestine relative to HM-fed piglets. Further studies are warranted to measure these outcomes more directly, to determine the magnitude of functional change occurring with differences in diet. Regardless, the current study highlighted that the type of infant diet can profoundly alter the bioregional microbiome and tissue transcriptome of piglets, which may have a variety of effects on gut and whole-body health.

## MATERIALS AND METHODS

### Study design and sample collection.

Animal maintenance and experimental treatments were conducted in accordance with the ethical guidelines for animal research established and approved by the Institutional Animal Care and Use Committee at the University of Arkansas for Medical Sciences. The experimental design for the current study has been previously described. Briefly, 48 2-day-old White Dutch Landrace Duroc male piglets were transferred from Metz Farm, Russellville, AR, USA, to individual housing at the vivarium of the Arkansas Children’s Nutrition Center, Little Rock, AR, USA. The individual housing allowed us to track the daily feed intake for each piglet during the experimental period. Piglets were randomly assigned into two groups: a human milk group (HM; *n* = 26; Mothers’ Milk Bank of North Texas, TX, USA) or a cow’s milk formula group (MF; *n* = 26) fed isocaloric diet (Similac Advance; Abbott Laboratories, Columbus, OH, USA) (see Table S1 in the supplemental material). They were fed an isocaloric diet and in accordance with the NRC nutrient and energy recommendations for growing piglets (10) from day 2 of age until weaning at day 21 of age. The piglets in the two groups were fed either HM or MF every 2 h during the first week of the study, every 4 h during the second week of study, and every 6 h during the third week of study to provide 1.047 MJ/kg of body weight per day. Diet composition and nutritional contents for HM and MF milk have been published previously (38) (Table S1 in the supplemental material). Solid starter feed was introduced from day 14 to day 21 of age. During the postweaning period, i.e., from day 21 to day 51 of age, piglets were fed solid starter feed *ad libitum* without milk supplementation in accordance with the NRC nutrient and energy recommendations for growing piglets (10). Eleven randomly selected piglets from each group were euthanized at day 21 of age, whereas the remaining piglets from each group (HM, *n* = 15; MF, *n* = 15) were euthanized at day 51 of age to collect ileal epithelium scrapings (EP) and ileal tissue (IL). Samples were immediately snap-frozen in liquid nitrogen and stored at −80°C for subsequent metagenome and transcriptome analyses.

### Ileum epithelial metagenome.

**(i) DNA extraction and shotgun metagenomic sequencing.** DNA was extracted from 100 mg of ilea mucosa using the QIAamp Fast Stool minikit protocol (Qiagen) following the manufacturer’s standard instructions. For each sample, total DNA concentration and purity were evaluated using a NanoDrop 2000c spectrophotometer (Thermo Fisher Scientific, USA) at wavelengths of 260 and 280 nm. Extracted DNA was immediately stored at –80°C. DNA libraries were prepared using an Illumina Nextera XT kit (Illumina, Inc., San Diego, CA, USA) followed by shotgun sequencing performed on an Illumina NextSeq 500 platform with a 2 × 150-bp, paired-end run. Raw sequence data files were demultiplexed and converted into FASTQ files using Casava v.1.8.2 (Illumina, Inc., San Diego, CA, USA).

**(ii) Shotgun processing of bacterial species and metabolic function profiling.** The FASTQ sequence files were uploaded to the Metagenome Rapid Annotation Using Subsystems Technology (MG-RAST) ver. 4 webserver to determine taxonomic composition and to predict the functional gene profiles (84, 85). Data representing the bacterial species and metabolic function profiles were obtained by annotating the query reads against RefSeq and SEED subsystem database tools implemented in MG-RAST, respectively (84, 85). The sequences are publicly accessible at the MG-RAST webserver (https://www.mg-rast.org) under accession identification numbers mgm4874995.3 to mgm4875042.3. The microbiome statistical analyses and visual explorations were performed using the Web-based tool MicrobiomeAnalyst (86, 87). The input tab-delimited plain-text files submitted to MicrobiomeAnalyst were operational taxonomic unit (OTU) tables containing read counts of bacterial species across the different samples from the same time point (either day 21 or day 51), a metadata file describing the group information for those samples, and a taxonomy table containing the taxonomic ranking for the bacterial species according to Greengenes Taxonomy (86). The uploaded data were filtered to remove low-quality features to enhance downstream statistical analyses (86). The default criteria for data filtration were used for low-count filtering (minimum count = 4, prevalence in samples = 20%) and for low-variance filtering (proportion to be removed = 10% based on interquartile range) (86). Data normalization was performed using the default data normalization criteria, including data scaling performed using total sum scaling (86). The differences in taxonomic composition at the bacterial phylum level between HM and MF samples were visualized using the default criteria for stacked bar/area plot option in MicrobiomeAnalyst (86). The differences in bacterial phyla and species relative abundances between the HM and MF groups were evaluated using the Mann-Whitney pairwise comparison test under the classical univariate analysis option in MicrobiomeAnalyst where significance was determined at a *P* value of ≤0.05 (86). Data representing the β-diversity between the HM and MF groups were computed using principal-coordinate analysis (PCoA) based on nonphylogenetic Bray-Curtis distance metrics and the nonparametric multivariate analysis of variance test (PERMANOVA) implemented in MicrobiomeAnalyst (86). The α-diversity was calculated in MicrobiomeAnalyst using observed species, Chao1, Shannon, and Simpson indices where the Mann-Whitney pairwise comparison test was applied to detect significant differences between the two groups at a *P* value of ≤0.05 (86). To analyze and visualize changes in the predictive functional profiles between HM and MF groups, Statistical Analysis of Metagenomic Profiles software (STAMP ver. 2.1.3) was used to assess and illustrate shifts in microbial functions using the two-sided Welch’s *t* test, where significance was determined at a *P* value of ≤0.05 (88).

**(iii) RNA-seq sample preparation.** Ileum tissue was subjected to RNA isolation using an miRNeasy kit from Qiagen following the manufacturer’s standard instructions. cDNA libraries were constructed using Illumina’s TruSeq stranded mRNA sample preparation kit according to the manufacturer’s protocol. Briefly, 500 ng of total RNA was subjected to poly(A) selection via the use of oligo(dT) to enrich for mRNA, chemically fragmented, and converted to single-stranded cDNA using the random hexamer-primed reverse transcription method. Second-strand synthesis was then performed to generate double-stranded cDNA, followed by fragment end repair and the addition of a single A base to each end of the cDNA. Combinatorial dual (CD) index adapters were then ligated to the fragment ends to enable attachment to the sequencing flow cell and sample pooling. Next, library DNA was PCR amplified and validated for fragment size and quantity using an Advanced Analytical Fragment Analyzer (AATI) and a Qubit fluorometer (Life Technologies, USA), respectively. Libraries concentrations were normalized, pooled, and then denatured and diluted to a final sequencing concentration of 1.8 pM. The diluted library was added to a NextSeq reagent cartridge (V2.0; Illumina, USA) for sequencing on a NextSeq 500 platform (Illumina, USA) using a high-output flow cell to generate approximately 25 million 75-base reads per sample. All sequencing was conducted by the Center for Translational Pediatric Research Genomics Core Lab at Arkansas Children’s Research Institute (Little Rock, AR, USA).

**(iv) RNA-seq data analysis.** RNA reads were checked for quality of sequencing using FastQC v.0.11.7 (89). The adaptors and low-quality bases (*Q* < 20) were trimmed to a minimum of 36 bp using Trimmomatic v0.36 (90). Reads that passed quality control were aligned to the Sus scrofa 11.1 (Ensembl release 91; GenBank Assembly identifier [ID] GCA_000003025.6) reference genome using TopHat v2.1.1 (91), and gene-level expression counts were determined using the htseq-counts (92) module implemented in Blast2GO v5.1.13 (93). Only reads uniquely aligned to known genes were retained and counted. Genes with low counts were then removed before downstream analysis was performed. To retain the maximum number of interesting features, genes with a minimum of 1 cpm in at least 11 libraries at day 21 and 3 libraries at day 51 were retained for further investigation. The filtered data sets were then normalized for compositional bias using the trimmed mean of M values (TMM) (94, 95). EdgeR’s quasi-likelihood method [glmQLFTest()] was then used to identify genes that were differentially expressed between experimental groups (96–100). Results determined for genes with a false-discovery-rate (FDR)-corrected *P* value of 0.05 and a fold change (FC) value of >2 were considered statistically significant (101). The transcriptome data were then used to generate a volcano plot (log_2_ FC versus log_10_ negative *P* value) to display differentially expressed genes (DEGs) in the MF group compared with the HM group using OriginPro, Ver. 2019b (OriginLab Corporation, Northampton, MA, USA). Pathway enrichment analysis was performed using Ingenuity Pathway Analysis software (IPA; Ingenuity Systems, Redwood City, CA, USA) to distinguish the top 20 canonical pathways in which the differentially expressed genes identified in the MF group were enriched based on the highest −log(*P* value). Fisher’s exact test was used to compute a *P* value that denoted the probability of the differentially expressed genes in the pathway being found together due to random chance.

### Data accessibility.

The metagenome data are publicly accessible at the MG-RAST webserver (https://www.mg-rast.org) under accession identification numbers mgm4874995.3 to mgm4875042.3.
