# Supplementary material for: Formula Diet Alters the Ileal Metagenome and Transcriptome at Weaning and during the Postweaning Period in a Porcine Model
Source: mSystems. 2020 Aug 4;5(4):e00457-20. doi: 10.1128/mSystems.00457-20 (PMC7406227; doi:10.1128/mSystems.00457-20)
Supplement: TABLE S1 [file mSystems.00457-20-st001.docx]

**Supplemental TABLE S1** Diet Composition of milk formula, human milk, and sow milk^1^

| **Nutrient** | **Milk formula** | **Human milk** | **Sow milk** |
| --- | --- | --- | --- |
| \| **Macro nutrients** \| \| --- \| \| Protein^2,3^ \| \| Carbohydrate \| \| Fat^4,5^ \| \| **Essential amino acids** \| \| Arginine \| \| Histidine \| \| Isoleucine \| \| Leucine \| \| Lysine \| \| Methionine \| \| Phenylalanine \| \| Threonine \| \| Trytophan \| \| Valine \| \| **Non-Essential amino acids** \| \| Cystine \| \| Tyrosine \| \| Alanine \| \| Aspartic Acid \| \| Glutamic Acid \| \| Glycine \| \| Proline \| \| Serine \| \| **Minerals** \| \| Calcium^2,6^ \| \| Phosphorous^2,7^ \| \| Sodium^2,8^ \| \| Chloride^8,9^ \| \| Magnesium^10^ \| \| Potassium^2,7^ \| \| Copper^11^ \| \| Iodine^11^ \| \| Iron^12^ \| \| Manganese^11^ \| \| Selenium^11^ \| \| Zinc^11^ \| \| **Vitamins** \| \| Vit A (all-trans retinol)^13^ \| \| Vit D (cholecalciferol)^13^ \| \| Vit E (RRR-α-tocopherol)^13^ \| \| Vit K (phyloquinone)^13^ \| \| Biotin^13^ \| \| Choline^13^ \| \| Folic acid^13^ \| \| Nicotinamide^13^ \| \| Pantothenic Acid^13^ \| \| Riboflavin^13^ \| \| Thiamin^13^ \| \| Vit B6 (pyridoxine)^13^ \| \| Vit B12 (cyanocobalamin)^13^ \| | \|  \| \| --- \| \| 53827 \| \| 59579 \| \| 73000 \| \|  \| \| 2514 \| \| 1706 \| \| 3660 \| \| 8034 \| \| 6352 \| \| 1462 \| \| 3331 \| \| 3199 \| \| 1341 \| \| 3409 \| \|  \| \| 2074 \| \| 2521 \| \| 3213 \| \| 7036 \| \| 10767 \| \| 1114 \| \| 3225 \| \| 2831 \| \|  \| \| 3000 \| \| 3100 \| \| 1100 \| \| 1500 \| \| 150 \| \| 4200 \| \| 3.2100 \| \| 0.10 \| \| 51.00 \| \| 1.6200 \| \| 0.14 \| \| 50.7500 \| \|  \| \| 1.61 \| \| 0.0115 \| \| 6.21 \| \| 0.21 \| \| 0.045 \| \| 240 \| \| 0.188 \| \| 12.73 \| \| 6.85 \| \| 2.21 \| \| 1.16 \| \| 1.12 \| \| 8 \| | \|  \| \| --- \| \| 54500 \| \| 67440 \| \| 69000 \| \|  \| \| 2579 \| \| 1691 \| \| 3745 \| \| 8320 \| \| 6448 \| \| 1383 \| \| 3283 \| \| 3153 \| \| 1424 \| \| 3384 \| \|  \| \| 2332 \| \| 2708 \| \| 3458 \| \| 7600 \| \| 11164 \| \| 1218 \| \| 3305 \| \| 2867 \| \|  \| \| 2800 \| \| 3000 \| \| 1100 \| \| 1500 \| \| 150 \| \| 4100 \| \| 2.9000 \| \| 0.16 \| \| 40.48 \| \| 1.6000 \| \| 0.15 \| \| 47.0000 \| \|  \| \| 1.86 \| \| 0.0022 \| \| 3.45 \| \| 0.17 \| \| 0.024 \| \| 254 \| \| 0.179 \| \| 7.97 \| \| 5.90 \| \| 1.64 \| \| 0.79 \| \| 86.10 \| \| 7 \| | \|  \| \| --- \| \| 56000 \| \| 48000 \| \| 78000 \| \|  \| \| 3250* \| \| 1230* \| \| 2410* \| \| 4590* \| \| 4190* \| \| 770* \| \| 1980* \| \| 2010* \| \| 873 \| \| 2860* \| \|  \| \| 828 \| \| 2148 \| \| 1533 \| \| 4353 \| \| 12264 \| \| 1264 \| \| 6042 \| \| 2965 \| \|  \| \| 1630** \| \| 1390 \| \| 430 \| \| NA \| \| 100 \| \| 590*** \| \| 0.0013 \| \| NA \| \| 2.00** \| \| 0.0001 \| \| NA \| \| 0.0063 \| \|  \| \| 0.96** \| \| 0.0093 \| \| 1.70 \| \| 0.09 \| \| 0.015 \| \| NA \| \| 0.003 \| \| 7.40*** \| \| 4.00*** \| \| 1.80*** \| \| 0.69 \| \| NA \| \| NA \| |

^1^Expressed as mg/L diet. In addition to powdered formula and human breast milk include added nutrients to meet energy and nutrient recommendations of the NRC for growing pigs. Formula based diet: Similac Advance Powder (Ross Products Abbott Laboratories, Columbus, OH). Human breast milk based diet: Human breast milk (Mothers' Milk Bank of North Texas, Fort Worth, TX). 0.27 g xanthum gum added as stabilizing agent.

^2^Whey Protein Isolate (WPI 895, Fonterra USA, Rosemont, IL) 47 g to formula based diet and 53 g to human breast milk based diet. Contains (mg/g basis) 900 protein, 5.6 sodium, 0.8 calcium, 0.5 potassium, 0.4 phosphorus.

^3^Amino Acid Mix 2.14 g added to formula based diet and human breast milk based diet. Amino acids were purchased (Sigma Aldrich, St. Louis, MO) and mixed before addition to diets. Contains (mg/g basis) 330 L-Arginine, 210 L-Histidine, 460 L-Phenylalanine.

^4^LouAna Safflower oil (LouAna, Opelousas, LA) 25.6 g added to formula based diet and 22.4 g added to human breast milk based diet. Contains (mg/g) 143 polyunsaturated fat, 786 monounsaturated fat, 71 saturated fat.

^5^LouAna Coconut oil (LouAna, Opelousas, LA) 16.5 g added to formula based diet and 14.5 g added to human breast milk based diet. Contains (mg/g) 71 monounsaturated fat, 929 saturated fat.

^6^Calcium acetate (Sigma Aldrich, St. Louis) 10.0 g added to formula based diet and human breast milk based diet

^7^Potassium phosphate (Sigma Aldrich, St. Louis) 12.43 g added to formula based diet and human breast milk based diet

^8^Sodium chloride (Sigma Aldrich, St. Louis) 1.68 g added to formula based diet and human breast milk based diet

^9^Choline chloride (Sigma Aldrich, St. Louis) 0.26 g added to formula based diet and human breast milk based diet

^10^Magnesium acetate (mg/L) (Sigma Aldrich, St. Louis) 1.0 g added to formula based diet and human breast milk based diet

^11^Trace Mineral Mix. Minerals were purchased (Sigma Aldrich, St. Louis) and compounded before adding to diets. 0.22 g added to formula based diet and human breast milk based diet. Contains (mg/g) 920 zinc sulfate, 48 cupric sulfate, 0.4 potassium iodide, 26 manganese chloride, 2 magnesium selenate

^12^Iron sulfate (Sigma Aldrich, St. Louis) 0.2 g added to formula based diet and human breast milk based diet

^13^Vitamin Mix. Vitamins were custom compounded (Custom Compounding Center, Little Rock, AR) and added to diets. 1.13 mL added to formula based diet and human breast milk based diet. Contains (mg/mL) .429 retinyl acetate, .0015 cholecalciferol, 4.7 D-alpha tocopherol, 0.15 phytonadione, 0.02 biotin, 0.09 folic acid, 5.85 niacinamide, 3.76 dexpanthenol, 1.17 riboflavin, 0.53 thiamine, 0.68 pyridoxine hydrochloride, 0.00585 cyanocobalamin (μg/L).

*Huang, Y. and A. Sinclair. 1998. Lipids in Infant Nutrition. 1st ed. AOCS Publishing.

**Barnhart, C. E., D. Catron, and C. C. Culbertson. 1954. The Effect of Rations on Selected Vitamin Content of Sows' Milk. Journal of animal science 13(2):375-382.

***Kuiken, K. A. and P. B. Pearson. 1949. The essential amino acid (except tryptophan) content of colostrum and milk of the cow and ewe. The Journal of nutrition 39(2):167-176.

The values provided in sow milk column are based on the available published information, does not necessarily reflect NRC recommendations.
